# Robotic surgery for pelvic organ prolapse with complete bladder eversion

**DOI:** 10.1002/iju5.12522

**Published:** 2022-08-12

**Authors:** Manabu Ichino, Hitomi Sasaki, Masashi Takenaka, Keiichiro Ichihara, Akihiro Kawai, Kosuke Fukaya, Kenji Zennami, Kiyoshi Takahara, Makoto Sumitomo, Ryoichi Shiroki

**Affiliations:** ^1^ Department of Urology Fujita Health University School of Medicine Toyoake Japan

**Keywords:** pelvic organ prolapse, robotic surgery, urinary bladder eversion

## Abstract

**Introduction:**

Pelvic organ prolapse with complete bladder eversion is extremely rare.

**Case presentation:**

An 82‐year‐old woman was diagnosed with uterine prolapse 3 years ago and underwent occasional urethral catheter placement for difficulty in micturition. She presented with vulvar bleeding and prolapsed uterus from the vagina. Pelvic examination revealed uterine prolapse and a 65 × 65‐mm red mass ventrally with urinary outflow. Contrast medium leakage from the vulvar mass and guidewire observed on antegrade pyeloureterography indicated pelvic organ prolapse with complete bladder eversion. Manual reduction of the everted bladder, robotic sacrocolpopexy, and bladder neck reconstruction was performed. However, eversion recurred 10 months postoperatively. Subsequently, robotic Burch colposuspension, cystopexy to the rectus fascia, bladder neck reconstruction, colpoclesis, and cystostomy were performed. There was no recurrence postoperatively.

**Conclusion:**

Robotic Burch colposuspension, cystopexy to the rectus fascia, bladder neck reconstruction, colpoclesis, and cystostomy were performed for complete bladder eversion.

**Abbreviations & Acronyms**
POP: Pelvic organ prolapse
QOL: quality of life
S–Cr: serum–creatinine

**Keynote message**

Complete bladder eversion with concurrent uterine prolapse has been rarely reported and can significantly affect a patient's quality of life. This report presents our case of a patient in whom sacrocolpopexy and bladder neck reconstruction with complete bladder eversion were followed by a recurrence 10 months postoperatively. Additional management using the robotic Burch colposuspension technique was necessary. The clinical implication is that sarcrocolpopexy and bladder neck reconstruction alone may not be sufficient for the management of pelvic organ prolapse with complete bladder eversion.

## Introduction

Complete bladder eversion is rare, and studies reporting cases of complete bladder eversion with concurrent uterine prolapse are scarce. Herein, we report a case of pelvic organ prolapse (POP) with complete bladder eversion, managed successfully using the robotic Burch colposuspension technique.

## Case presentation

An 82‐year‐old woman (gravida 1, para 1) was diagnosed with uterine prolapse 3 years ago. She also had difficulty in micturition, for which a urethral catheter was occasionally inserted for 6 months. She visited our hospital due to vulvar bleeding and difficulty in manually reducing the prolapsed uterus.

### Pelvic examination

**Fig. 1:**
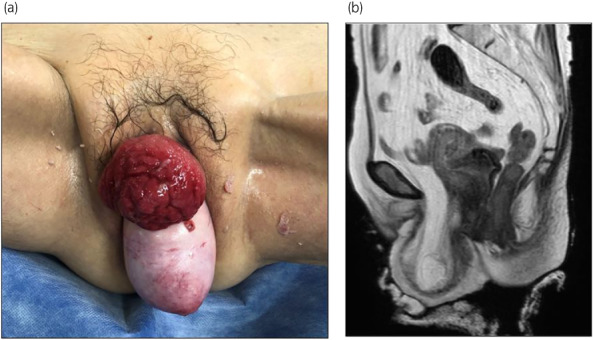
(a) Pelvic examination at the first visit. Legend: Uterine prolapse and protrusion of a red mass on its ventral side. (b) Magnetic resonance imaging at the first visit (T2‐weighted image). Legend: Bladder lumen could not be confirmed.

Pelvic examination revealed uterine prolapse with protrusion of a red‐colored mass (65 × 65 mm) on its ventral side (Fig. 1). Although identifying the external urethral meatus was difficult, urinary outflow from the mass was detected, suggesting bladder eversion from the external urethral meatus.

### Blood biochemical analysis

Biochemical blood analysis revealed the presence of severe inflammatory reaction (white blood cell count, 17.7 × 103/μL; C‐reactive protein, 5.22 mg/dL) and decreased renal function (serum blood urea nitrogen, 36.4 mg/dL; serum–creatinine [S–Cr], 2.68 mg/dL).

### Diagnostic imaging

**Fig. 2:**
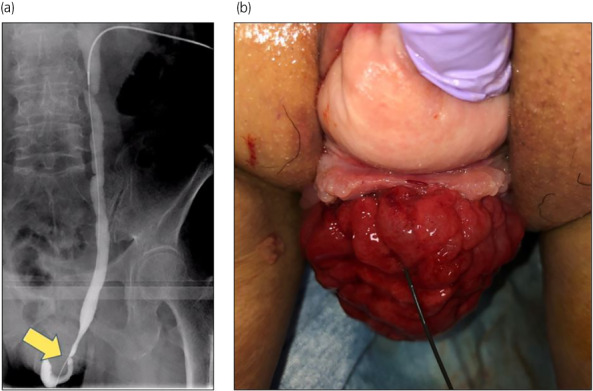
(a) Antegrade pyeloureterography. (b) Pelvic examination at antegrade pyeloureterography. A guide wire was inserted downward antegradely to confirm the left ureteral orifice.

Computed tomography revealed bilateral hydroureteronephrosis. Magnetic resonance imaging showed prolapse of adipose tissue into the retropubic space, but the urinary bladder lumen could not be confirmed (Fig. 1b). Antegrade pyeloureterography (Fig. 2) and ureteral stent placement were performed to improve renal function. Leakage of the contrast medium and the guidewire were observed in the red vulval mass (Fig. 2b); thus, complete bladder eversion was diagnosed.

#### Initial treatment and progress

Robotic supracervical hysterectomy and sacrocolpopexy and bladder neck reconstruction were performed. Bladder neck reconstruction involved trimming the edge of the dilated bladder neck, dividing it into two layers, leaving 10 mm on the ventral side, and suturing using a polyglactin suture. A Foley catheter was placed from the bladder neck.

Postoperatively, bilateral hydronephrosis disappeared, and the S–Cr level improved to 1.4 mg/dL. Cystography performed 2 months postoperatively revealed a bladder capacity of 80 mL and no granulation formation. Subsequently, the cystostomy catheter was removed, resulting in total urinary incontinence. However, complete bladder eversion recurred 10 months later. According to DeLancey's theory,^1^ the level of bladder eversion is divided into levels 1, 2, and 3 depending on the position of the pelvic organ supporting tissue. The reason for the recurrence was considered to be that complete bladder eversion was a level 3 (pelvic floor muscle) weakness and the sacral fixation was dislodged. We chose to perform robotic Burch colposuspension and robotic sacrocolpopexy concurrently; we considered that a rating of DeLancey level 3 required anatomical repair of complete bladder eversion.

#### Second treatment and progress

The bladder was completely everted and prolapsed completely from the vagina. The bladder neck was dilated to 35 mm; the everted bladder was manually reduced from the perineum.

**Fig. 3:**
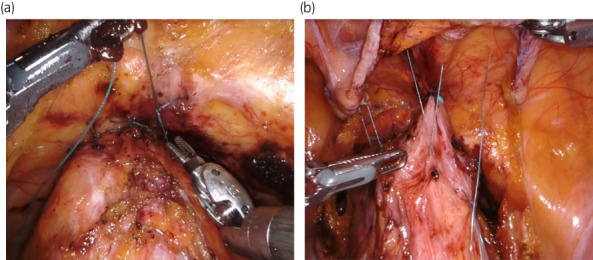
(a) Robotic Burch colposuspension. (b) Cystopexy to the rectus fascia. Surgical procedure.

The da Vinci Xi™ was used for robotic surgery. The abdominal cavity was observed using a laparoscope, and the mesh was confirmed to be fixed to the sacral anterior surface. The anterior bladder cavity was opened, and the anterior surface of the urinary bladder and posterior surface of the pubis was sutured at 10 points using polyester sutures (Fig. 3a), and cystopexy was performed to the rectus fascia at five points using polyester sutures (Fig. 3b). Transvaginal surgery was performed for bladder neck reconstruction using the same procedure as the previous surgery. In addition, colpoclesis and cystostomy were performed. The operative time was 4 h 12 min, the robotic console operative time was 2 h 53 min, and the blood loss was 5 mL. Postoperatively, a Foley catheter was not placed from the bladder neck. The postoperative course was favorable, and the patient was discharged 10 days after surgery. Cystostomy enabled postoperative urination management, and there was no recurrence of complete bladder eversion 12 months postoperatively.

## Discussion

Complete bladder eversion is an extremely rare condition wherein the urinary bladder prolapses from the female external urethral orifice in an everted state.^2^ Only 11 patients have been reported since 2000, 8 of whom had concurrent uterine prolapse (Table 1). Complete bladder eversion is thought to be caused by a combination of the following factors: (i) fragility of urethral tissues due to decreased elasticity of the vaginal wall and urethra and decreased blood flow to the urethral mucosa associated with postmenopausal estrogen deficiency; (ii) enlargement of the urogenital hiatus due to untreated uterine prolapse, rendering the supporting tissues between the pubis and urethra/bladder fragile; (iii) chronic increase in abdominal pressure due to difficulty in micturition; and (iv) infection around the urethra and stimulation of urethral tissues due to placement of a urethral catheter.^2^, ^3^, ^4^ The following risk factors for the development of complete bladder eversion have been reported: (i) long‐term placement of a urethral catheter; (ii) sexual activity via the urethra; and (iii) susceptibility of the urethra to serious infections occurring in the adjacent tissues and other factors (malignant tumor, pelvic organ prolapse, trauma, difficulty in urination, increased abdominal pressure due to labor, advanced age, and menopause).^2^, ^3^, ^4^ In this patient, the urethral catheter was present for a long time to ease the difficulty in micturition caused by the POP; therefore, these risk factors were applicable. The aim of complete bladder eversion treatment is to restore the pelvic floor anatomically and physiologically while maintaining the normal genitourinary function, improving the postoperative quality of life (QOL), and ensuring long‐term efficacy. Fixation of the urinary bladder to the rectus abdominis fascia and vaginal closure is performed in conjunction. However, since most patients with this condition are elderly, in whom the rectus abdominis muscle may be fragile, recurrence may occur. The pathological conditions in our patient included POP with complete bladder eversion and bilateral hydronephrosis, resulting in postrenal renal failure. Therefore, we decided that mesh treatment, such as sacrocolpopexy, was the best option. Robotic sacrocolpopexy was performed; however, the condition recurred 10 months later. Anatomically, complete bladder eversion results from vulnerabilities of DeLancey level 3, suggesting that Burch surgery should have been the first treatment of choice. Regarding postoperative urination management, there have been many reported cases of urinary tract diversion, such as cystostomy, instead of spontaneous urination.^2^, ^3^, ^5^, ^6^, ^7^ Although urinary tract diversion might have reduced the postoperative QOL, it was considered the best option for urination management; the possibility of recurrence was minimal.

**Table 1:** Reported case of complete bladder eversion

Year	Author	Age	Multiparity	Concurrent uterine prolapse	Predisposing factors	Ureteral obstruction	Treatment
2021	Present case	81	G1 P1	Yes	Foley catheter indwelling	Bilateral	Robotic supracervical hysterectomy and sacrocolpopexy, Bladder neck reconstruction →Robotic Burch Colposuspension, Cystopexy to the rectus fascia, Bladder neck reconstruction, Colpoclesis, Cystostomy
2019	Mae Delara^5^	89	Not reported	Yes	Not reported	No	Bladder neck closure, Perineoplasty, Cystostomy
2018	Yamamichia^6^	81	G3 P3	Yes	Pessary	Bilateral	Vaginal hysterectomy, Cystopexy to the rectus fascia, Colpoclesis, Perineoplasty, Bladder neck closure, Cystostomy
2010	Raffi^7^	73	Not reported	Yes	Foley catheter indwelling	Bilateral	Colpoclesis, Bladder neck closure, Cystostomy
2010	Kim^2^	75	G4 P4	Yes	Pessary, Foley catheter indwelling	Bilateral	Vaginal hysterectomy, Colpocleisis, Cystostomy Bladder neck reinforcement with retropubic prolene mesh
2010	Lowe G^8^	46	Not reported	Yes	Hemipelvectomy	Not reported	Abdominal hysterectomy and sacrocolpopexy, Bladder neck closure and creation of Monti catheterizable stoma
2009	Kalorin^3^	76	G7 P7	No	Foley catheter indwelling	Yes	Cystopexy to anterior abdominal wall, Cadaveric fascia suburethral sling, Cystostomy
2007	Acharya^9^	72	Not reported	Yes	Foley catheter indwelling	Not reported	Transurethral reduction
2006	Kim^10^	78	G10 P8	No	Bladder Cancer	Bilateral	Radical cystectomy and ileal conduit for adenocarcinoma
2004	Dunn^11^	78	G12 P12	No	Pessary, Vesicovaginal fistula	Bilateral	Transurethral reduction, Fistula repairing
2002	Mastropietro^4^	Not reported	Yes	Yes	Foley catheter indwelling	No	Vaginal hysterectomy, Colpocleisis, Levator ani muscle plication, Suburethral plication, Cadaveric fascial suburethral sling

## Conclusion

Robotic sacrocolpopexy and bladder neck reconstruction were performed for POP with complete bladder eversion; however, eversion recurred 10 months later. Therefore, robotic Burch colposuspension, cystopexy to the rectus fascia, colpoclesis, and cystostomy were performed. There was no recurrence postoperatively. Currently, urination is managed via cystostomy. In the future, we are considering the removal of the Foley catheter and urination management by total incontinence.

## Author contributions

Manabu Ichino: Writing – original draft. Hitomi Sasaki: Supervision. Masashi Takenaka: Writing – review and editing. Keiichiro Ichihara: Writing – review and editing. Akihiro Kawai: Writing – review and editing. Kosuke Fukaya: Writing – review and editing. Kenji Zennami: Writing – review and editing. Kiyoshi Takahara: Writing – review and editing. Makoto Sumitomo: Writing – review and editing. Ryoichi SHIROKI: Supervision.

## Conflict of interest

The authors declare no conflict of interest.

## Ethical approval

Not applicable.

## Approval of the research protocol by an Institutional Reviewer Board

Not applicable.

## Informed consent

Not applicable.

## Registry and the Registration No. of the study/trial

Not applicable.
